# A Comparison of Pathogenic Eukaryotic Elongation Factor 2 (EEF2) Variants in Spinocerebellar Ataxia 26 Versus De Novo Mutations

**DOI:** 10.7759/cureus.26857

**Published:** 2022-07-14

**Authors:** Hongfei Zhao, Nikolas Mata-Machado

**Affiliations:** 1 Pediatric Neurology, University of Illinois at Chicago, Chicago, USA

**Keywords:** pediatric neurology, case report, eef2 gene, spinocerebellar ataxia 26, spinocerebellar ataxia

## Abstract

In this article, we present an eight-year-old boy with developmental delay and cerebellar symptoms who was found to have a de novo eukaryotic elongation factor 2 (EEF2) mutation on genetic testing. Previously, pathogenic mutations in EEF2 have been associated with spinocerebellar ataxia 26. An extremely rare disorder, spinocerebellar ataxia type 26 (SCA26), has only been documented as an autosomal dominant, inherited, late-onset ataxia in one six-generation family of Norwegian descent. However, three pediatric patients with de novo EEF2 mutations were recently discovered, presenting with noncerebellar symptoms such as syndactyly, developmental delay, and behavioral issues. The patient of this study was found to have features similar to both adult patients with SCA26 as well as previous pediatric patients with de novo mutations.

## Introduction

Spinocerebellar ataxias (SCA) are a diverse group of autosomal dominant ataxias resulting from a variety of genetic mutations, many of which are yet to be identified. In fact, over 25% of patients diagnosed with SCA have mutations in previously unknown genomic locations [1]. Patients present with progressive ataxia are often accompanied by other symptoms such as oculomotor dysfunction, dysarthria, dysmetria, tremor, and pyramidal and cortical impairment [1]. Many of the more common SCAs are the result of trinucleotide repeats; these ataxias typically present later in life, although anticipation resulting in progressively larger expansions can cause symptoms to manifest earlier. Other SCAs, however, are the result of single mutations in several identified locations on the genome. For instance, spinocerebellar ataxia type 26 (SCA26) is the result of a eukaryotic elongation factor 2 (EEF2) gene mutation, located on chromosome 19p13.3 [2]. The resulting disruption in translocation during protein synthesis is believed to result in an eventual loss of Purkinje cells, leading to pure cerebellar ataxia with cerebellar-specific atrophy [3]. Pathologic mutations in the EEF2 gene have been identified in several generations of a single family as well as in three pediatric patients with de novo mutations [4]. The patient in this report is, to the author’s knowledge, the fourth documented pediatric patient with a pathologic de novo EEF2 mutation.

## Case presentation

The patient is an eight-year-old male who presented with speech and motor developmental delay, obesity, attention-deficit/hyperactivity disorder (ADHD), and behavioral issues. He was vaginally delivered, and his mother's pregnancy was complicated by gestational diabetes, urinary infection, and decreased fetal movement toward the end of pregnancy. Concerns for fine and gross motor skill development include difficulty with writing, using scissors, and playing sports. He is bilingual, and his mother reports him having articulation difficulties in Spanish and English. At school, he struggles with inattentiveness and hyperactivity, and his teacher reports he has difficulty empathizing with his peers, becoming easily frustrated when playing with other children. He often becomes nervous or angry due to schedule changes, but he has never been formally evaluated for autism spectrum disorder (ASD). His mother notices he sometimes has staring episodes both at home and school, which she attributes to his ADHD. The patient endorses occasional blurry vision, but a recent optometry visit found no visual deficits. He complains of frequent itchiness and burning pain in the corners of his toes. Per the patient’s mother, he has a half-sister with similar developmental delays and a first-degree cousin with a learning disability, though neither of them has received neurologic workup for their symptoms. He was referred to a geneticist due to a concern for Prader-Willi syndrome and received whole-exome sequencing that revealed a heterozygous de novo variant of uncertain significance in the EEF2 gene (variant c. 2207 C>T, p. A736V). There was no evidence of Prader-Willi or fragile X syndrome.

Regarding his developmental history, he was breastfed until three years of age with no feeding difficulties. He first began walking at 18 months and was able to ambulate normally by two years of age. He spoke his first word at three years. He is currently in third grade, and his performance has been slightly below grade level. He received speech therapy/occupational therapy (ST/OT) services and is seeing a behavioral therapist. Other history includes macrosomia, epistaxis, adenotonsillectomy for obstructive sleep apnea, an appendectomy, and iron deficiency for which he takes 325 mg ferrous sulfate supplements. He is also on daily dexmethylphenidate 5 mg for ADHD.

On the physical, the patient is noted to be overweight (BMI in the 99.4th percentile) with a large stature for his age and features consistent with overgrowth; unfortunately, no head circumference was recorded in any visit. He has no distinctive facies. Notably, he possesses bilateral single transverse creases and fifth finger clinodactyly. His left fourth and fifth toes are short proximally and overlapping. The nails on the left fourth and fifth toes are curved. His strength and range of motion were unimpaired, though he walks with his right foot intermittently medially rotated. When asked to cross his legs while standing, he had balance issues. His tandem gait was mildly impaired, and he sways minimally on the Rhomberg. He also exhibited mild dysmetria in his finger-to-nose test on the left side. He had normal muscle tone and 2+ reflexes. His speech in English was not noticeably impaired, though he did appear to have difficulties maintaining attention, especially during the visual field test. On the vision exam, his responses were incorrect for his left eye on the lateral upper and lower fields. His horizontal smooth pursuit was impaired with no nystagmus.

**Figure 1:**
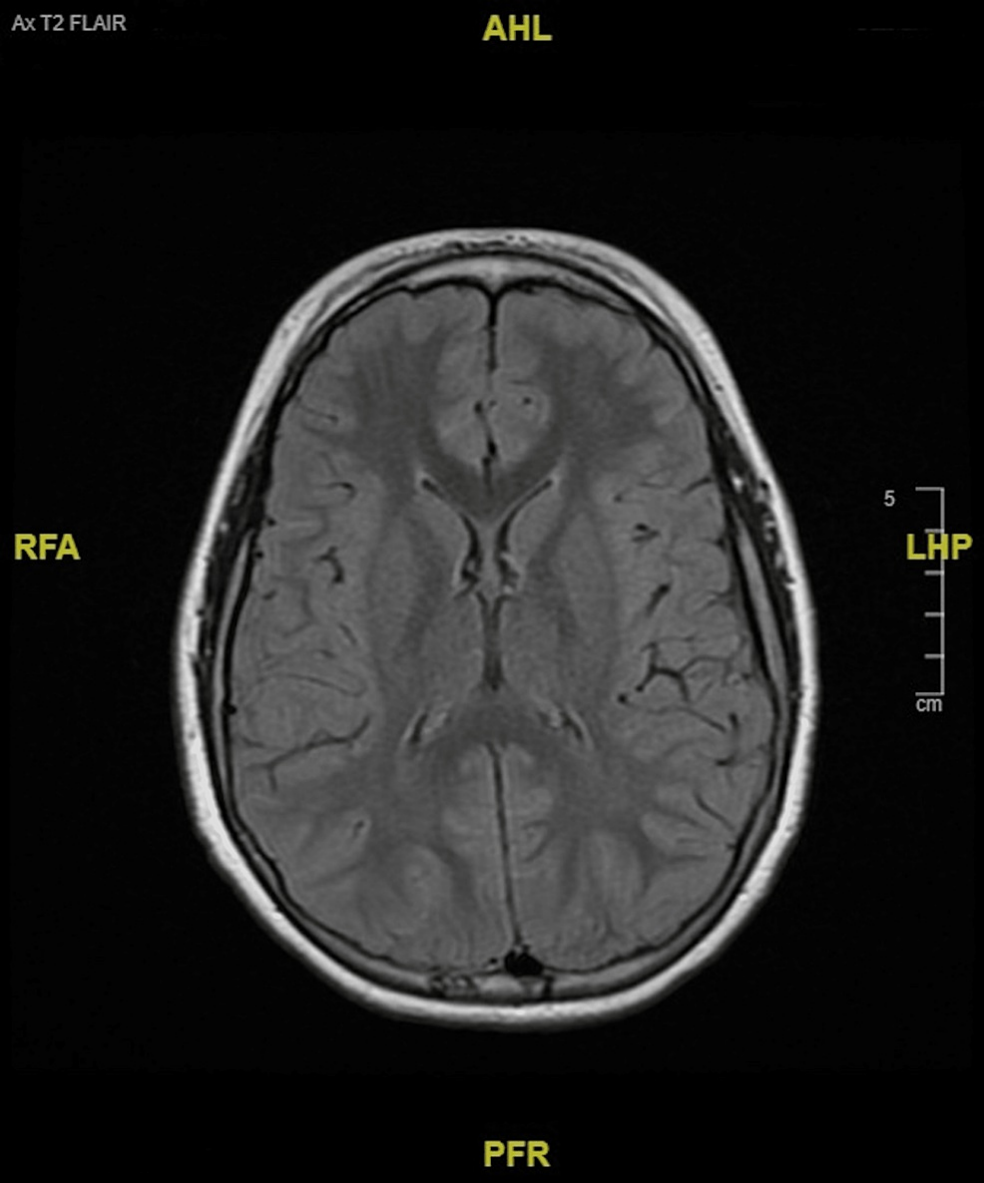
MRI brain shows normal ventricles The image shows a T2-weighted FLAIR sequence taken on a 1.5-Tesla scanner. Dotarem intravenous contrast (12 mL) was administered. MRI brain with contrast found no acute pathology. FLAIR: Fluid-attenuated inversion recovery.

**Figure 2:**
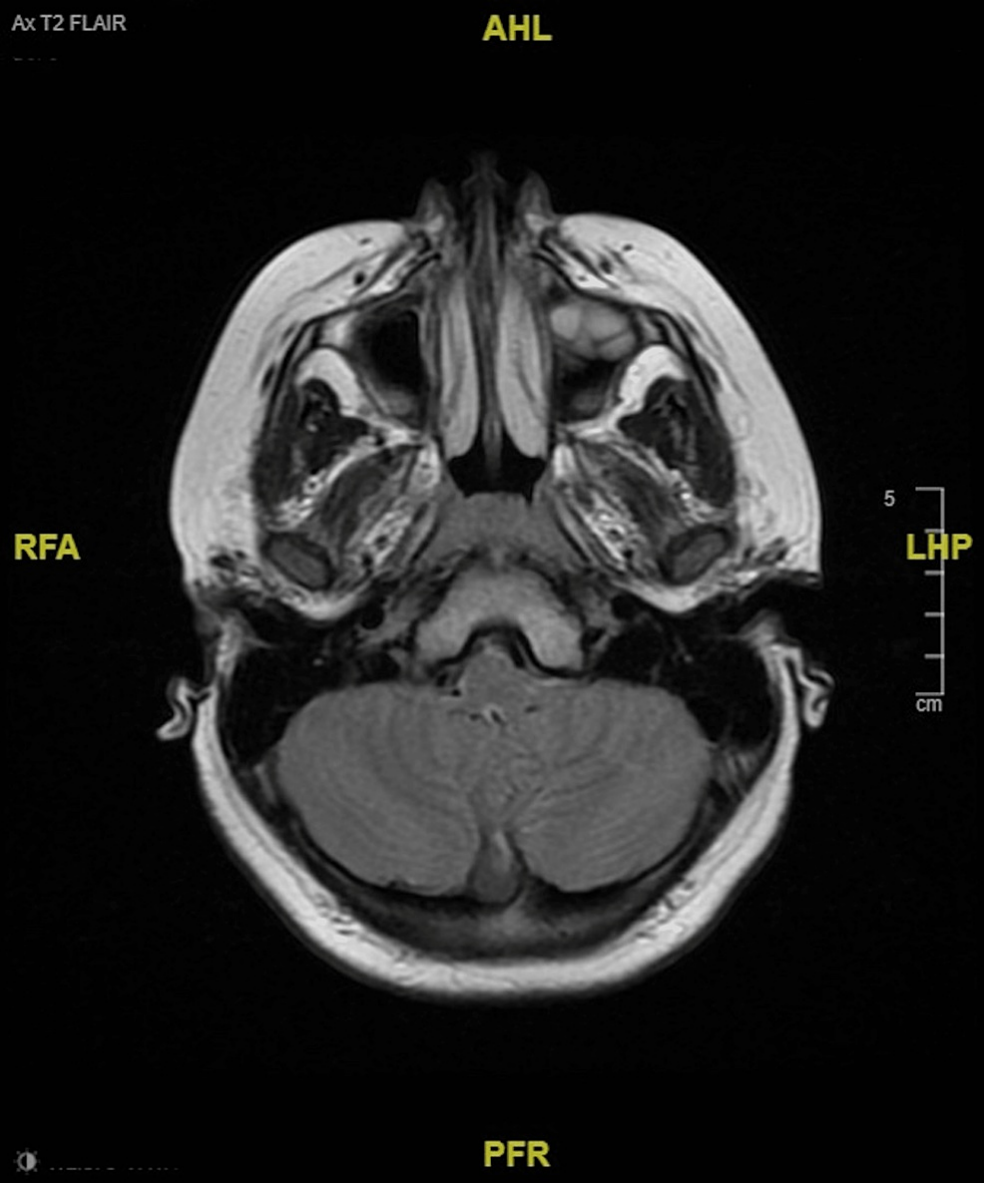
MRI brain shows normal cerebellum The image shows a T2-weighted FLAIR sequence taken on a 1.5-Tesla scanner. Dotarem intravenous contrast (12 mL) was administered. The cerebellar hemispheres have normal volume without evidence of signal change or mass effect. FLAIR: Fluid-attenuated inversion recovery.

**Figure 3:**
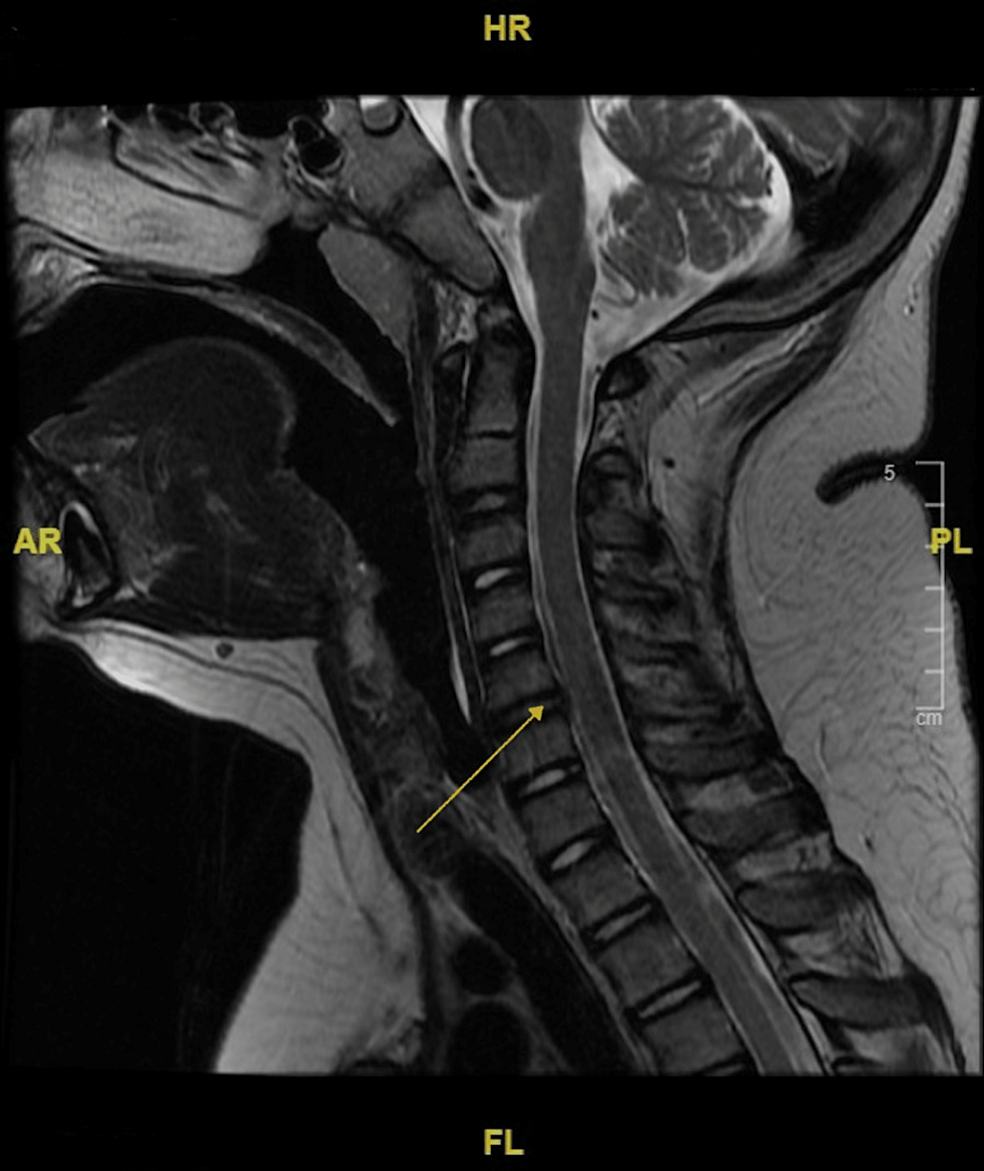
MR cervical spine with contrast shows mild C5-C6 disk bulge The image shows a T2-weighted FLAIR sequence taken on a 1.5-Tesla scanner. Dotarem intravenous contrast (12 mL) was administered using MR cervical spine protocols. There is a normal anatomic alignment of the cervical spine and no evidence of focal disk herniation or cord compression. The intervertebral disc heights and signals are maintained. FLAIR: Fluid-attenuated inversion recovery.

In other findings, the patient received a transthoracic echocardiogram (TTE) that revealed no congenital heart defects. An electroencephalogram (EEG) ordered to rule out the absence of seizures in the context of his staring episodes found no seizure activity. MRI with contrast was normal and visualized no pathology. There was slightly prominent retrocerebellar fluid without mass effect, and ventricles were within normal limits (Figures 1, 2). MR C-spine found a small C5-C6 disk bulge and a mildly slender cervical spinal canal, but the cord has normal contour, size, and signal (Figure 3).

## Discussion

SCA26 is an autosomal dominant pure cerebellar ataxia resulting from a pathogenic variant in the EEF2 gene. It was first discovered and studied in a six-generation family with Norwegian ancestry [2]. The 24 affected individuals were found via neurological exam and MRI abnormalities. These individuals presented with pure cerebellar signs, notably truncal and limb ataxia, gait ataxia, and impaired visual pursuit. The average age of onset was 42 years, with a range of 26-60 years, with no evidence of anticipation [5]. MRI found isolated cerebellar atrophy, with sparing of the pons and medulla [2]. The patients had no cognitive or intellectual deficits, no significant sensorimotor deficits, and no seizure disorders. This presentation is starkly different from the other pathologic variant in the EEF2 gene.

In a recent study from Oxford in 2020, three pediatric patients were identified as possessing missense variants in the EEF2 gene via peripheral blood samples [4]. The patients were all male (ages: three, six, and nine years), and they all presented predominantly with neurodevelopmental delays, with few to absent cerebellar signs. Rather than MRI abnormalities being confined to cerebellar atrophy as with the previously identified SCA26 individuals, the pediatric patients exhibited various structural brain abnormalities, including benign external hydrocephalus, with their lateral and third ventricles especially dilated. One patient also had diffuse thinning of his corpus callosum and left temporal-occipital focal dysplasia. All patients presented with speech and motor delays and digit malformation. Bilateral single transverse palmar creases, short fingers, and mild 2-3 toes syndactyly were found in one of the patients. He and another patient both possessed clinodactyly of the fifth finger. On their neurological exams, one patient was hypotonic with an unsteady gait and high stepping. Another exhibited poor motor coordination. Regarding vision abnormalities, one patient was myopic, and another had strabismus requiring surgery, but there were no documented issues with smooth pursuit. One patient experienced febrile seizures when he was 2.5 years old but had a normal EEG one month later. Behavioral problems similar to ASD were also found in one of the patients.

While both the late-onset SCA26 and the more recently discovered de novo mutations in pediatric patients result from a pathogenic EEF2 mutation, there are clear phenotypic differences between these two populations. For one, the three pediatric patients did not exhibit ataxia and cerebellar signs, despite all of them experiencing motor delays. Notably, visual pursuit in the de novo patients was not documented to have been impaired. In contrast, the pediatric patient of this study exhibited mild dysmetria in his left upper extremity (LUE) as well as an impaired smooth pursuit. However, patients with SCA26 had no symptoms unrelated to cerebellar dysfunction, including no speech delays, autistic behavior, MRI abnormalities outside of cerebellar degeneration, or dysmorphic features. Therefore, in most aspects, the patient of this study presents more similarly to the pediatric patients with the de novo mutations. He has bilateral single transverse creases, clinodactyly of the fifth finger, toes syndactyly, and behavioral abnormalities. His MRI notably depicted no cerebellar degeneration or atrophy, and his imaging, on the whole, was unremarkable. This is in line with the variable presentations on the MRIs of the other pediatric patients.

## Conclusions

Due to the small sample size of the de novo mutation patients, the lack of data about longitudinal disease progression, and the variable phenotypes among the four pediatric patients found, it is difficult as of yet to determine whether these patients could be classified as having an early-presenting variant of SCA26 or whether they should be diagnosed with a different genetic disorder altogether. In general, for de novo mutations of uncertain significance, we should restrain from connecting to syndromes prematurely. As in this case, many of the features of coordination and developmental delay could be unrelated. More information is required to determine whether these symptoms are connected, and longitudinal studies, in particular, would elucidate whether SCA26 and de novo EEF2 mutations are separate disorders or merely the same disease with differing severity due to age of onset.

## References

[1] Manto MU (2005). The wide spectrum of spinocerebellar ataxias (SCAs). Cerebellum.

[2] Yu GY, Howell MJ, Roller MJ, Xie TD, Gomez CM (2005). Spinocerebellar ataxia type 26 maps to chromosome 19p13.3 adjacent to SCA6. Ann Neurol.

[3] Hekman KE, Yu GY, Brown CD (2012). A conserved eEF2 coding variant in SCA26 leads to loss of translational fidelity and increased susceptibility to proteostatic insult. Hum Mol Genet.

[4] Nabais Sá MJ, Olson AN, Yoon G (2021). De novo variants in EEF2 cause a neurodevelopmental disorder with benign external hydrocephalus. Hum Mol Genet.

[5] Fujioka S, Sundal C, Wszolek ZK (2013). Autosomal dominant cerebellar ataxia type III: a review of the phenotypic and genotypic characteristics. Orphanet J Rare Dis.

